# Video-assisted retroperitoneal debridement for infected pancreatic necrosis: A single center series

**DOI:** 10.1016/j.ijscr.2022.107254

**Published:** 2022-06-02

**Authors:** Chih Ching Wu, David T. Martin, Brent D. Bauman, Stuart K. Amateau, Nabeel Azeem, James V. Harmon

**Affiliations:** a Medical School, University of Minnesota, Minneapolis, Minnesota, United States of America; b Department of Surgery, University of Minnesota, Minneapolis, Minnesota, United States of America; c Division of Gastroenterology, University of Minnesota, Minneapolis, Minnesota, United States of America

**Keywords:** Acute necrotizing pancreatitis, Infected necrotizing pancreatitis, Video assisted retroperitoneal debridement, Open pancreatic necrosectomy, Step-up approach

## Abstract

**Introduction and importance:**

Open anterior necrosectomy is no longer considered the first approach for the up to 20% of patients with acute pancreatitis who develop infected necrosis. When endoscopic debridement and percutaneous drains fail, video assisted retroperitoneal debridement (VARD) is now the favored surgical approach.

**Case presentation:**

We report a current patient who underwent the VARD procedure and provide a seven-year follow-up on ten additional patients who underwent the VARD procedure for infected pancreatic necrosis between 2010 and 2015. We analyzed patient demographics, APACHE II scores, length of hospital stay, number of VARD procedures, and surgical complications.

**Clinical discussion:**

During this study period, we cared for 320 patients, 59 women, and 261 men, with acute necrotizing pancreatitis. Ten of our patients ultimately underwent the VARD procedure. We report a 50% overall complication rate and a 20% mortality rate. Two of the patients who underwent the VARD procedure had already undergone open necrosectomy; two additional patients in the VARD group required open necrosectomy after the VARD procedure.

**Conclusion:**

Our current case report demonstrates the effectiveness of the VARD procedure for patients with infected pancreatic necrosis. Our case series provides additional details regarding the significant morbidity and mortality associated with infected pancreatic necrosis. We acknowledge that despite following the step-up protocol, both VARD procedures and open necrosectomy may still be required.

## Highlights

• VARD is a first option when surgery is required for infected necrotizing pancreatic necrosis.
• The VARD procedure requires placement of percutaneous CT guided retroperitoneal flank drains.
• Complications of the VARD procedure include bleeding, bowel injury, and pancreatic fistula.

## 1 Introduction

Acute necrotizing pancreatitis is associated with severe abdominal pain that may require advanced endoscopic, radiologic, or surgical intervention. Necrotizing pancreatitis is defined when 30% or more of the pancreatic parenchyma is necrotic. The mortality for patients with sterile necrotic pancreatitis can be as high as 10%; mortality for patients with infected pancreatic necrosis has been reported to be as high as 30% [1]. A study of the mechanism of acute pancreatitis suggests that an overwhelming excess of calcium and trypsinogen activation play a role in the pathophysiology of necrotizing pancreatitis [2]. Lysosomal Cathepsin B and Nuclear factor-κB have also been implicated in the pathogenesis of acute necrotizing pancreatitis [2], [3], [4]. Typically acute necrotic collections (ANC) are recognized within the first 3–4 weeks following the onset of symptoms. Generally, walled off necrosis (WON) can be recognized after an additional 3–4 weeks Both ANC and WON can be either sterile or infected. Patients with infected pancreatic necrosis nearly always require debridement procedures.

When percutaneous or internal debridement procedures have failed, video assisted retroperitoneal debridement (VARD) procedures have replaced the need for classical anterior open necrosectomy. Nearly all pancreatic centers have implemented the step-up approach for the management of necrotizing pancreatitis starting with minimally invasive procedures for debridement of the WON [5]. Minimally invasive options include endoscopic transluminal necrosectomy, sinus tract endoscopy for debridement, and the VARD procedure representing the final element of the step-up approach. The benefits of the step-up approach for patients with infected pancreatic necrosis were confirmed in a randomized controlled trial of the VARD procedure versus the traditional anterior open necrosectomy [5]. In that study, patients managed by the step-up approach demonstrated a reduced incidence of organ failure, diabetes, and incisional hernias.

We report our experience with patients diagnosed with infected pancreatic necrosis at a single academic center. We discuss the clinical and postoperative outcomes following the VARD procedure in our single-center case series. Our enhanced VARD technique replaces the anterior open surgical necrosectomy which would be required in the rare patients who fail to recover using the step-up approach.

## 2 Methods

We performed a retrospective review of all patients with a seven-year follow-up who underwent the VARD procedure for infected pancreatic necrosis at our academic medical center. We also present a current patient case report. Demographics, age, sex, BMI, APACHE II score, length of hospital stay, number of VARD procedures, and outcome were reviewed and analyzed. Our retrospective review was approved by the institutional review board at the University of Minnesota (IRB number 1611M99281). Our case series have been reported in line with the SCARE 2020 criteria [6].

We hold weekly interdisciplinary meetings attended by surgical and gastrointestinal medicine faculty to review patients' clinical progress, analyze imaging studies, and determine the optimal minimally invasive, radiologic, or surgical approach. The anatomic location of necrotic collections determines whether endoscopic internal drainage or percutaneous drainage will be most advantageous. Internal endoscopic drainage and debridement procedures are guided by transgastric or transduodenal endoscopic ultrasound (EUS). The preferred route for percutaneous drainage is via the left flank into the left retroperitoneum space for suitable collections; anterior percutaneous drain placement should be avoided. Gerota's fascia, the colon, the spleen, the kidney, and major blood vessels serve as critical anatomic landmarks during the placement of percutaneous left flank retroperitoneal drains [7]. These left flank CT-guided percutaneous drains enter the retroperitoneum between the descending colon and the left kidney. Right flank CT guided percutaneous drains enter the retroperitoneum between the ascending colon and the right kidney. Small 8 Fr percutaneous drains are usually upsized to 12–14 Fr drains before initiating the VARD procedure [7], [8]. Source control requires adequate drainage of infection to decrease the risk of ongoing sepsis [7].

**Fig. 1:**
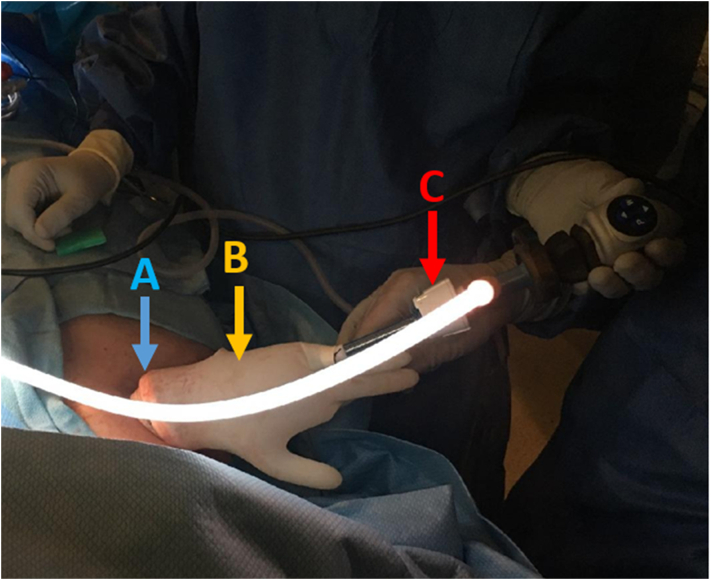
Intraoperative photo of right flank VARD procedure. A.) Deployment of the wound protector (blue arrow). B.) Utilization of a sterile glove (yellow arrow) to accommodate multiple trocar placement. C.) Single 12 mm trocar (red arrow) for laparoscope, light source, and camera through one finger of the sterile glove during the VARD procedure. (For interpretation of the references to colour in this figure legend, the reader is referred to the web version of this article.)

Minimally invasive endoscopic or percutaneous interventions preceded all VARD surgical procedures. These percutaneous CT-guided retroperitoneal flank drains serve as a guide to performing the VARD procedure. An incision is made sharply at the percutaneous flank drain site to approach retroperitoneal necrotic collections. The drain is followed as it enters the abdominal wall musculature. The retroperitoneum is entered bluntly. Several methods are available to explore the retroperitoneum. A 15 mm laparoscopic trocar can be placed along this tract and CO2 insufflation applied. A 5 mm laparoscope and another laparoscopic instrument can be inserted in tandem through this large port. A 6 cm incision also facilitates the placement of a small dual-ring wound protector (Applied Medical, Rancho Santa Margarita, CA). The application of a sterile surgical glove around this wound protector permits the deployment of multiple 5 mm laparoscopic instruments maintaining retroperitoneal inflation using the wound protector (Fig. 1). The incision can alternatively be enlarged to 10 cm and the retroperitoneal space can be further evaluated using lighted breast flap handles and ring forceps to extract large collections of necrotic pancreatic tissue. However, this loses the benefits of CO_2_ insufflation. If retroperitoneal hemorrhage is encountered, the wound can be tightly packed and if needed, Resuscitative Endovascular Balloon Occlusion of the Aorta (REBOA) can be used to control major hemorrhages encountered during the VARD procedures. At the completion of the VARD procedure, a 19 Fr round Blake drains is placed deep into the cavity and secured at the level of the skin incision, which is loosely re-approximated with surgical clips. If significant bleeding is present, the wound may be packed with Kerlix gauze and washed out, and re-explored within 1–2 days. Once bleeding is controlled, a 19 Fr round Blake drain is placed and the skin edges are re-approximated. CT scans are typically obtained 5–7 days following the VARD procedure to assess the resolution of necrotic pancreatic collections, or as clinically indicated. If septic shock continues, or if a large necrotic pancreatic collection persists, VARD procedures can be repeated.

## 3 Patient case report

**Fig. 2:**
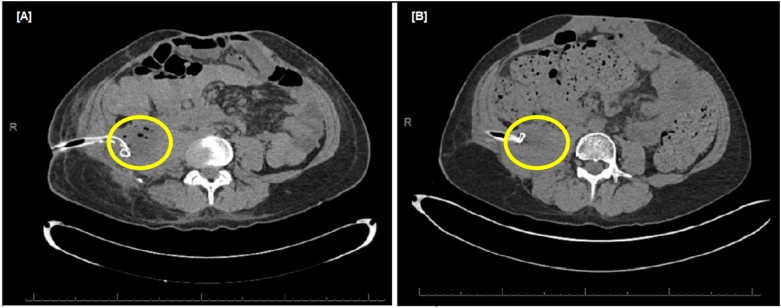
[A] CT scan shows percutaneous drain in right retroperitoneal infected collection (yellow ellipse) prior to VARD. [B] Resolution of the abscess cavity (yellow ellipse) with drain in place following VARD procedures. (For interpretation of the references to colour in this figure legend, the reader is referred to the web version of this article.)

We report a 41-year-old man who underwent video assisted retroperitoneal debridement (VARD) procedures for the management of infected pancreatic necrosis. The patient developed necrotizing pancreatitis following cholecystectomy and endoscopic retrograde cholangiopancreatography (ERCP) at a referring hospital. The patient was transferred to our academic medical center and underwent emergent anterior decompressive laparotomy for the management of abdominal compartment syndrome. The anterior abdominal wall was reconstructed with bridging vicryl mesh and skin grafts, and the patient was discharged to a long-term care facility. Two months later, the patient returned with ongoing infected pancreatic necrosis, and the VARD procedure approach was selected as it was particularly advantageous due to the patient's mesh and abdominal skin grafts. Abdominal computed tomography (CT) scans below and after the VARD procedure demonstrate the resolution of the infected pancreatic collection (Fig. 2). *Staphylococcus aureus*, *Pseudomonas aeruginosa*, *Finegoldia magna*, and *Candida tropicalis* were isolated from the necrotic material. Two percutaneous drains were placed resulting in the eventual complete resolution of the patient's necrotic collections.

## 4 Results

We report a seven-year follow-up for 320 patients with necrotizing pancreatitis cared for at our academic medical center. Ultimately, ten patients, 5 male, and 5 female, underwent the VARD procedure when minimally invasive endoscopic and percutaneous treatment options failed. The average age was 41 years (range 24–71 years) and the average BMI was 34 (range 21–51) for the patients undergoing the VARD procedure. Table 1 provides additional information regarding the patients' demographics, comorbidities, and etiology of pancreatitis. The length of hospital stay ranged from 8 to 197 days. The median APACHE II Score of our population was 19. Two patients had an anterior necrosectomy before the VARD procedure and two patients had an anterior necrosectomy after the VARD procedure. The observed mortality was 20% (Table 2). One patient had a postoperative myocardial infarction, two patients developed *C. difficile* colitis, two patients developed pneumonia, and one patient developed a splenic abscess. Table 3 reports the postoperative complications, infections, and mortality. Patients in this study underwent an average of 2.4 VARD procedures and required a median hospital stay of 58 days. Table 4 provides additional information regarding patients' characteristics and postoperative outcomes.

**Table 1:** Patient demographics: age, sex, BMI, comorbidities, and etiology of pancreatitis. Comorbidities: renal failure, cancer, neuromuscular disorders, obesity, and congestive heart failure. Etiology of pancreatitis: alcohol related, biliary, hypertriglycemia, or iatrogenic.

Patient	Age	Sex	BMI	Pre-op morbidity	Etiology
1	24	F	21	Respiratory failure	Alcohol Related
2	24	M	38	Respiratory failure, sepsis, renal transplant	Hyperlipidemia
3	37	M	33	Metastatic testicular cancer	Post ERCP
4	40	M	27	Neuromuscular disorder	Gallstone pancreatitis
5	46	F	51	Renal failure, morbid obesity	Gallstone pancreatitis
6	31	F	36	Renal failure septic shock	Alcohol related
7	42	M	32	Seizure disorder	Gallstone pancreatitis
8	63	M	29	Respiratory failure, renal failure, shock	Idiopathic
9	38	F	37	Respiratory failure, septic shock, pneumonia,	Hypertriglyceridemia
10	71	F	35	CHF, renal failure, Shock	Gallstone pancreatitis

**Table 2:** Number of VARD procedures, anterior necrosectomy, length of hospital stay, and readmissions. Anterior necrosectomy prior to the VARD procedure (2). Laparotomy following VARD (2). Thirty-day readmission (2).

Patient	VARD procedures	Anterior necrosectomy (Y/N)	Length of hospital stay	30 day readmission (Y/N)
1	4	N	52	N
2	1	N	57	Y
3	3	N	8	Y
4	2	Y (prior to VARD)	47	N
5	5	N	62	N
6	3	Y	59	Expired
7	2	Y (prior to VARD)	89	N
8	7	Y	197	Expired
9	1	N	167	N
10	1	N	51	N

**Table 3:** Post-operative complications, infections, and mortality. Myocardial infarction (1), renal failure (1), *C. diff* colitis (2), pneumonia (2), splenic abscess (1), and expired patients. (2).

Patient	Myocardial infarction (Y/N)	Renal failure (Y/N)	Post op infection	Mortality (Y/N)
1	N	N	C. diff colitis	N
2	N	N	N	N
3	N	N	N	N
4	N	N	N	N
5	N	N	N	N
6	N	N	N	Y
7	N	N	Pneumonia	N
8	Y	N	Splenic abscess, Pneumonia	Y
9	N	Y	N	N
10	N	N	C. diff colitis	N

**Table 4:** Mean and median values for patients who underwent the VARD procedure. SD = standard deviation. IQR = interquartile range.

Variable	Value (mean, median, [IQR])
Age (mean/SD)	41 years ± 15 years
Female	50%
BMI (mean/SD)	34 ± 8
APACHE II score (median, [IQR])	19 [10,29]
Number of VARD procedures (median, [IQR])	2.4 procedures [1,4]
Length of hospital stay (median, [IQR])	58 days [50,108]
Predicted mortality (median, [IQR])	26% [12,56]
Observed mortality	20%

## 5 Discussion

Our case report and case series demonstrate the outcomes of the VARD procedure first described by Horvath et al. as an alternative to anterior abdominal necrosectomy [9]. We applied the step-up approach and the VARD procedure rather than performing an anterior necrosectomy which is now the standard of care as established by the PANTER trial [5]. We selected candidates for the VARD procedure based on hemodynamically stability, resolution of coagulopathy, and the absence of abdominal catastrophe such as bowel perforation, ruptured pancreatic pseudoaneurysm, or expanding peripancreatic hematomas. We made the diagnosis of acute necrotizing pancreatitis using clinical information, laboratory data, and imaging studies. We prefer to avoid early CT scans with intravenous contrast until patients have received adequate fluid resuscitation. Once CT scans are performed, the lack of contrast enhancement within the pancreas establishes the extent of pancreatic necrosis [10], [11], [12]. Although the use of MR imaging has recently been reported, we did not pursue this imaging technique in our patient series [10]. We provided fluid resuscitation, IV antibiotic therapy, nasal-jejunal enteric nutritional support, and pain control for all patients in our series. As supported in the literature, we typically initiate minimally invasive interventions for WON at approximately four weeks [11]. Minimally invasive drainage procedures are typically effective in resolving sepsis and early multiorgan failure in the vast majority of patients with infected pancreatic necrosis [7]. However, when patients fail to recover despite procedures, we advocated initiating the VARD procedure, an approach proven by Hartwig et al. to significantly decrease postoperative mortality [13].

Although evidence-based clinical practice guidelines recommend the application of the step-up approach and the VARD procedure, this surgical procedure is also associated with complications. Four patients in our series developed postoperative infectious complications. Two patients developed *C. diff* colitis; two patients developed postoperative pneumonia; one patient developed a splenic abscess. Two patients developed noninfectious postoperative complications; one patient suffered a myocardial infarction and another patient developed acute renal failure. (Table 3) Other investigators report similar complication rates associated with the VARD procedure including perioperative hemorrhage and colonic perforation [7], [8], [14]. To avoid postoperative infectious complications, we have instituted strict pulmonary physiotherapy protocols and adherence to judicious antibiotic stewardship. Collaboration with our surgical critical care team has also been enhanced to reduce noninfectious postoperative complications in patients undergoing the VARD procedure.

Although not seen in our patient series, other complications of the VARD procedures may occur. Postoperative persistent sinus tract drainage may develop following the removal of surgical drains; this typically resolves without further surgical intervention. Splenic vein thrombosis, commonly associated with acute pancreatitis, may also be seen in patients who undergo the VARD procedure [15]. Thrombosis of the middle colic artery and vein is also associated with acute pancreatitis and may result in acute colonic ischemia, colon perforation, and colocutaneous fistulas following the VARD procedure. Iatrogenic injury to the descending colon may result from percutaneous drainage procedures or the VARD procedure. Colonic perforations may occur after interventional radiology embolization to control bleeding in patients with infected pancreatic necrosis [16]. Although colonic fistulas may be seen in the perioperative period, these typically resolve without further intervention; if the patient demonstrates ongoing sepsis or peritonitis, a diverting colostomy may be indicated [17].

Two patients in our case series failed to improve following VARD procedures and required open anterior necrosectomy; both of these patients died resulting in a 20% mortality rate in our series. This mortality rate is similar to those reported in the literature [8], [14]. Conversion rates to open anterior necrosectomy of greater than 30% have been reported by other investigators [8]. The indications to convert to open necrosectomy include intraoperative hemorrhage, failure to achieve adequate debridement, and colonic perforation [14].

## 6 Conclusion

The step-up approach is supported by randomized clinical evidence and has become the standard of care for patients with necrotic pancreatic collections. We report our experience using the VARD procedure as a part of the step-up approach. Patients in our case series had a perioperative complication rate of 50% and a 30-day readmission rate of 20%. The 20% mortality rate in our series was associated with complex and severely ill patients who underwent the VARD procedure at our academic medical center. Our surgical program benefits from weekly interdisciplinary clinical working meetings attended by surgical faculty members, advanced pancreaticobiliary gastrointestinal medicine faculty members, and trainees from both disciplines.

## Abbreviations

VARD: video assisted retroperitoneal debridement
ANC: acute necrotic collection
WON: walled off necrosis
CT: computed tomography
CHF: congestive heart failure
EUS: endoscopic ultrasound

## Provenance and peer review

Not commissioned, externally peer-reviewed.

## Availability of data and materials

Not applicable.

## Sources of funding

This research did not receive any specific grant from funding agencies in the public, commercial, or not-for-profit sectors.

## Ethical approval

Our retrospective review was approved by the institutional review board at the University of Minnesota (IRB number 1611M99281).

## Consent

Our case report patient has provided written consent for case report publication. All surgical patients provided informed consent for surgery. Our retrospective review was approved by the institutional review board at the University of Minnesota (IRB number 1611 M99281).

Written informed consent was obtained from the patient for publication of this case report and accompanying images. A copy of the written consent is available for review by the Editor-in-Chief of this journal on request.

## Author contributions

James Harmon conceived of and supervised the project, wrote the original draft, participated in data acquisition and statistical analysis, and reviewed and edited the final manuscript.

Chih Ching Wu wrote the original draft and reviewed and edited the final manuscript.

Brent Bauman participated in data acquisition and statistical analysis and reviewed and edited the final manuscript.

David Martin reviewed and edited the final manuscript.

Stuart Amateau reviewed and edited the final manuscript.

Nabeel Azeem reviewed and edited the final manuscript.

## Research registration

N/A.

## Guarantor

James Vail Harmon, MD, PhD.

## Declaration of competing interest

The authors have no competing interests to disclose.
